# The effects of repeated whole genome duplication events on the evolution of cytokinin signaling pathway

**DOI:** 10.1186/s12862-018-1153-x

**Published:** 2018-05-29

**Authors:** Elisabeth Kaltenegger, Svetlana Leng, Alexander Heyl

**Affiliations:** 1 Department Biochemical Ecology and Molecular Evolution, Botanical Institute, Christian-Albrechts-University, Kiel, Germany; 2 Institute of Applied Genetics, Freie Universität Berlin, Berlin, Germany; 3 Biology Department, Adelphi University, Garden City, USA

**Keywords:** Gene duplication, Whole-genome duplication, Gene retention, Gene dosage balance, Cytokinin signaling, Signal transduction, Evolution

## Abstract

**Background:**

It is thought that after whole-genome duplications (WGDs), a large fraction of the duplicated gene copies is lost over time while few duplicates are retained. Which factors promote survival or death of a duplicate remains unclear and the underlying mechanisms are poorly understood. According to the model of gene dosage balance, genes encoding interacting proteins are predicted to be preferentially co-retained after WGDs. Among these are genes encoding proteins involved in complexes or in signal transduction.

**Results:**

We have investigated the way that repeated WGDs during land plant evolution have affected cytokinin signaling to study patterns of gene duplicability and co-retention in this important signal transduction pathway. Through the integration of phylogenetic analyses with comparisons of genome collinearity, we have found that signal input mediated by cytokinin receptors proved to be highly conserved over long evolutionary time-scales, with receptors showing predominantly gene loss after repeated WGDs. However, the downstream elements, e,g. response regulators, were mainly retained after WGDs and thereby formed gene families in most plant lineages.

**Conclusions:**

Gene dosage balance between the interacting components indicated by co-retention after WGDs seems to play a minor role in the evolution of cytokinin signaling pathway. Overall, core genes of cytokinin signaling show a highly heterogeneous pattern of gene retention after WGD, reflecting complex relationships between the various factors that shape the long-term fate of a duplicated gene.

**Electronic supplementary material:**

The online version of this article (10.1186/s12862-018-1153-x) contains supplementary material, which is available to authorized users.

## Background

Duplications of individual genes and whole genomes are a dominant feature of plant evolution and have been detected in all land plant lineages [1–4]. Gene duplication is assumed to be a stochastic process and a common fate of a duplicate is its loss [5, 6]. However, the retention of duplicate genes seems to be biased toward certain functional classes of genes [7–9]. Another factor that seems to influence the long-term survival of a duplicated gene is the mode of duplication as genes which are predominantly retained differ between whole-genome duplications (WGDs) and small-scale duplication events [10–14]. Studies of WGD events and their effect on core angiosperm genes (i.e., gene families shared by all angiosperm species) showed a generalized pattern of “gene duplicability”, meaning the ability of genes to be retained following WGD. Three categories could be defined: *a*) “singleton” genes: the majority of core genes occur as single copies and are functionally involved in the maintenance of genome integrity; *b*) “multicopy” genes: genes remain in a duplicated state throughout time and are functionally biased toward signaling, transport, and metabolism; and *c*) “intermediate” genes: these genes show a pattern of prolonged duplicate retention spanning several tens of millions of years following WGD but appear eventually to return to singleton status. This later group (intermediate genes) is enriched for genes that are involved in development, growth, and regulation of transcription [9].

For the categories *b*) and *c)*, gene dosage balance (GDB) theory is discussed to be a major driver of gene retention after WGD [9]. Basically, the GDB theory states that, for many genes whose products participate in protein complexes, the stoichiometry among interacting gene products (i.e., proteins) must be maintained [10, 15–18]. Thus, according to GDB, dosage-balance-sensitive genes are predicted to be co-retained after a WGD event. These genes are also predicted to continuously experience purifying selection after duplication leading to prolonged retention. This prolonged retention accompanied by the gradual circumvention of dosage-balance-constraints may increase the possibility that duplicate genes diversify (sub- or neofunctionalization) and become permanently preserved [17, 19]*.* Genes in the “multicopy” group may have been retained – at least initially – because of dosage balance constraints. The “intermediate” group of gene families can be explained by a scenario of dosage balance that wears off over time, leading to prolonged preservation but ultimate loss of duplicates [9]*.*

GDB has been extended to informational pathways (e.g., signal tansduction) [20], in agreement with the observation that preferentially retained gene categories after WGD include signal transduction genes in diverse species such as banana [21], *Arabidopsiss* [22], and the ciliate *Paramecium tetraurelia* [23]. Here, we have studied the pattern of gene retention and loss of the individual components of core cytokinin signaling after repeated WGDs during land plant evolution to test whether a bias exists in the gene duplicability of the individual components and to explore whether GDB can explain the observed pattern. Cytokinins are plant hormones that play pivotal roles in plant development and its response to changes in the environment [24]*.* Various studies have indicated that the cytokinin signaling system was established in early divergent land plants, and even some Charophyceae green algae have been found to encode family members of all four components of this signaling pathway [25–27]. Thus, cytokinin signaling is an ideal model system for studying the way that the independent and repeated WGDs during land plant evolution have affected the evolution of the individual components of a signaling pathway.

**Fig. 1:**
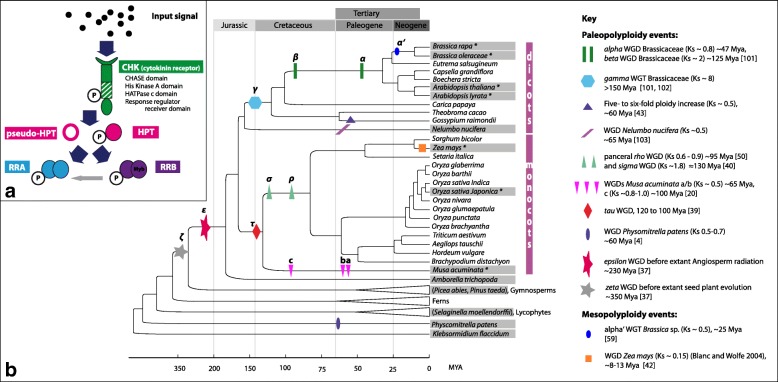
Cytokinin signaling and repeated polyploidy events during land plant evolution. **a** Schematic representation of core cytokinin signaling. Cytokinin receptors perceive cytokinins, autophosphorylate and transmit the signal via HPTs to RRAs and RRBs. Pseudo-HPTs may compete for phosphotransfer with HPTs. RRAs are induced by cytokinins and function as negative regulators to form feedback regulatory loops. RRBs encode DNA-binding transcription factors that mediate cytokinin-dependent transcriptional activation [24]. **b** Repeated WGDs and WGTs during land plant evolution and sampling strategy [4, 21, 37, 40, 41, 43, 44, 50, 95, 101, 102]. The figure illustrates the phylogenetic tree topology for land plants [93, 94, 96, 97]. *Klebsormidium flaccidum* is placed on the basal lineage of current land plants marking the transition from the aquatic to the terrestrial life form [25]. Ancestral polyploidy events in seed plants and angiosperms are indicated by symbols and were inferred from the literature, given in the key. Gray boxes mark the 14 core species chosen for this study of comparative analyses of cytokinin signaling (Table 1). For all depicted species/lineages, genes encoding CHKs were identified and their evolutionary history was reconstructed. Additionally, the evolutionary history of HPTs, RRAs, and RRBs from species labeled with * was reconstructed

The core signaling of the phytohormone cytokinin is mediated via a variant of the two-component signaling system [28] (Fig. 1a). The cytokinin molecules are perceived by binding to the Cyclases/Histidine kinases Associated Sensing Extracellular (CHASE) domain of a membrane-bound hybrid histidine kinase (CHASE domain containing histidine kinase, CHK) that serves as receptor [29, 30]. The binding of the hormone leads to the autophosphorylation of the histidine kinase domain. After an intramolecular phosphotransfer to the c-terminal response regulator domain of the receptor, the signal is transferred to histidine phosphotransfer proteins (HPTs). These proteins have been shown to shuttle between the cytoplasm and the nucleus [31]. The HPTs can be divided into enzymatically active and inactive orthologs (pseudo-HPTs). The pseudo-HPTs lack a conserved histidine residue that acts as a phosphorylation site and negatively interfere with pathway activity [32, 33]. HPTs can phosphorylate the response regulator domain of various response regulators. In cytokinin signaling, two types of response regulators have been shown to be important: *i*) the type-B response regulators (RRB), which are Myb type transcription factors that, upon phosphorylation, initiate the transcription of their target genes, and *ii*) the type-A response regulators (RRA), which are transcriptionally regulated by the RRB [34] and have been shown to be negative regulators of the cytokinin signaling pathway [35].

The study presented here reveals that the individual components of cytokinin signaling were duplicated and retained independently of each other. Although the cytokinin signaling pathway expanded mainly via WGD events, the observed pattern of gene duplicability and the pattern of co-retention after WGDs does not correlate with the predictions of GDB. Instead, downstream elements of the pathway show a trend towards higher gene duplicability compared with upstream elements.

## Results

### Repeated WGDs during land plant evolution provide the background to study the evolutionary patterns of the cytokinin signaling components

In order to study the evolutionary pattern of the individual components of cytokinin signaling after whole genome duplications, plant species were chosen for further analysis to cover the major meso- and paleopolyploidy events reported in land plant evolution [4, 36–38] (Fig. 1b). Furthermore, to allow the identification of all members of the four protein families involved in cytokinin signaling pathway the availability of a large dataset, e.g., a fully sequenced genome or transcriptome, was another criterion to select species. Thus, this study focused on 14 “core” plant species (Table 1, Fig. 1b) for comparative analyses of cytokinin signaling. Beginning with *Klebsormidium flaccidum* as a representative of the Charophyceae, the algae lineage that gave rise to land plants, the whole spectrum of land plants was covered.

**Table 1:** Copy numbers of the cytokinin signaling components

Species	CHKs	HPTs (*AHPT*/*PHPT*)	RRAs	RRBs
*Arabidopsis lyrata*	3	6 (5/1)	10	15
*Arabidopsis thaliana*	3	6 (5/1)	10	15
*Brassica oleracea*	4	8 (7/1)	15	19
*Brassica rapa*	4	8 (7/1)	20	20
*Nelumbo nucifera*	4	8 (6/2)	7	11
*Musa acuminata*	8	10 (8/2)	14	14
*Oryza sativa Japonica Group*	4	5 (2/3)	13	10^a^
*Zea mays*	7	5 (4/1)	12	9
*Amborella trichopoda*	2	4 (3/1)	4	6
*Picea abies*	2	7 (5/2)	12	7
*Pinus taeda*	3	3 (3/0)	13	9
*Selaginella moellendorffii*	2	2 (2/0)	3	10
*Physcomitrella patens*	11	2 (2/0)	7	5
*Klebsormidium flaccidum*	9	1 (1/0)	3	1

*Abbreviations:* *AHPT* authentic His-containing phosphotransfer protein, *PHPT* pseudo His-containing phosphotransfer protein, which lacks the conserved His

^a^without EHD1 copies: OrysatHPT25 OsRR27 and OrysatHPT24 OsRR30, including B-IV group, B-V group excluded (classification according to Tsai et al. [45])

### Comparison of copy numbers of the various cytokinin signaling components among the investigated species

Sequences encoding the four components of the cytokinin signaling pathway were identified in the core species and categorized as *bona fide* CHKs, HPTs, RRAs, or RRBs. The copy numbers of the identified components varied between species and also between the different protein families (Table 1). The number of cytokinin receptors was relatively stable between species, ranging from two to four copies across most land plants. Exceptions found were *M. acuminata*, *Zea mays*, *Physcomitrella patens*, and *K. flaccidum* with eight, seven, eleven, and nine receptor genes, respectively. In contrast, for HPTs, a steady increase in copy number was detected during land plant evolution starting from two HPTs in the moss *P. patens* to eight and 10 in *Brassica* species and *Musa acuminata*, respectively. In the gymnosperm *Picea abies,* seven HPT copies were identified, in comparison with three HPT copies in the closely related *Pinus taeda*. Another noteworthy trend was the emergence of pseudo HPTs in *P. abies* and in angiosperms with copy numbers ranging from one to two, with the exception of rice for which three pseudo HPTs were identified.

RRA and RRB copy number increased steadily during land plant evolution to form middle size gene families in dicots and monocots. Furthermore, with a few exceptions, the number of RRAs and RRBs in flowering plant species were found to be roughly equal (Table 1).

These differences in the copy number between the four gene families involved in cytokinin signaling indicated that the individual components experienced different evolutionary pressures that influenced their duplicability or rather their retention after WGD.

### Reconstruction of cytokinin receptor evolution

The complete sequence of cytokinin receptors included four protein domains (PFAM domains of CHASE, HisKinaseA, HATPase, response regulator receiver) as alignable regions, which covered in total 466 amino acids. To reconstruct CHK evolution during land plant evolution, genes encoding CHKs of the 14 above-mentioned “core species” (Table 1, Fig. 1) were analyzed. Additional species were sampled to improve the phylogenetic reconstructions (Fig. 1). Thus, the final dataset included CHKs from 51 plant species ranging from mosses, lycophytes, ferns, and gymnosperms up to flowering plants (Additional file 1: Table S1). To test the robustness of the tree topology, trees based on different substitution models (nucleotide, codon, and protein substitution models) were calculated and compared. Furthermore, maximum likelihood (ML) and Bayesian reconstruction were performed.

In the resulting trees, the main branching pattern was highly similar. Robinson-Foulds-(RF)-distances (Additional file 2: Table S2) between pairs of trees showed that less than 25% of branches were dissimilar for most pairwise comparisons. Codon models fitted the data best according to a model test with ModelOMatic [39]. Overall, phylogenetic signal in the dataset was sufficient, and tree topology was robust. All reconstructed trees supported the presence of three major clades within angiosperm CHKs (Additional file 3: Figure S1 and S2). These clades were named according to the three well-characterized cytokinin receptors of *A. thaliana*, which were located within the clades (the AHK2, the AHK3, and the AHK4 clade, respectively). The more basal branches of the phylogenetic tree reconstructions were generally less supported. Furthermore, the positioning of the CHKs from gymnosperms was inconsistent in the various tree reconstructions. However, CHKs of the early diverging land plants (lycophytes and bryophytes) fell in all tree reconstructions reproducibly outside the above-mentioned groups of the land plant CHKs. At the very base of the tree, we found sequences encoding CHKs of the algae *K. flaccidum* and the moss *P. patens*.

### Ancient duplications of cytokinin receptors

**Fig. 2:**
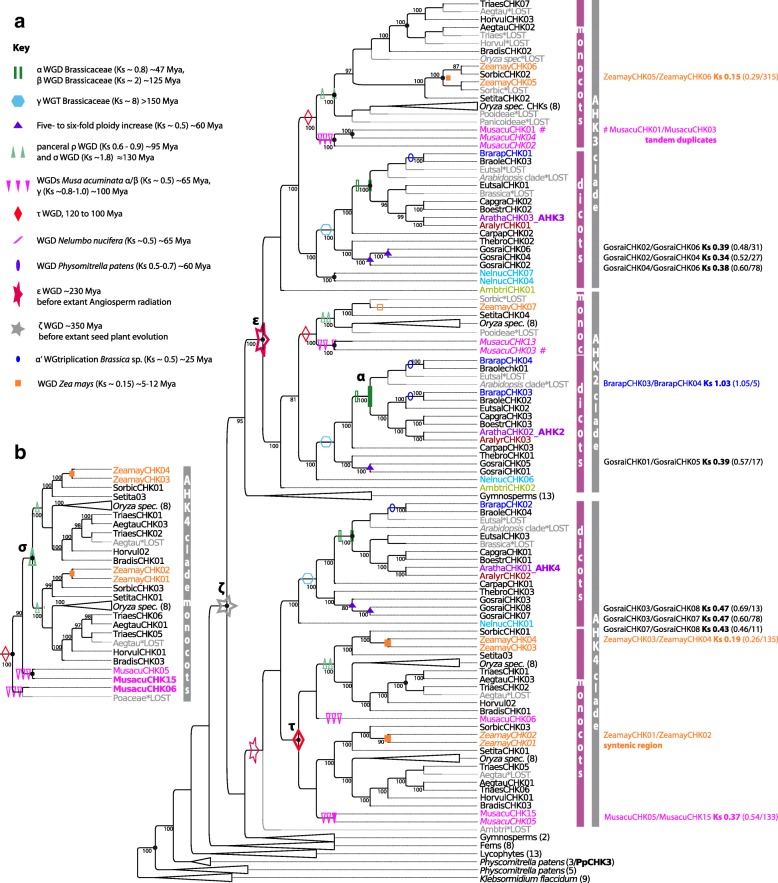
Reconciled tree of CHK encoding sequences. **a** Reconciled Bayesian tree of CHK encoding sequences (codon substitution model). Gene tree reconciliation includes rearrangement of branches with a support of less than 70% (posterior probability). Branch support of > 70% is given in the tree. Original Mr. Bayes tree see. Additional file 3: Figure S2. The optimization criterion for reconciliation was the number of duplications and losses. The three dominant clades of CHKs are indicated. Duplicates inferred to be lost are labeled (*LOST) and are given in gray. Of note, most duplicates resulting from WGDs that were lost thereafter were not detected. Thus, WGD events that occurred during CHK evolution are indicated with empty symbols (see key) on the corresponding branches, if resulting duplicates were not retained. If, according to phylogenetic inference plus syntenie/collinearity relationships, a copy resulting from a WGD duplication was retained, duplication nodes are labeled with the corresponding filled symbols, and Ks distances between the gene pairs are given. Furthermore, the median Ks, and the number of gene pairs of the total collinear region is given in brackets. Further duplications inferred from phylogenetic analysis are labeled with a black dot, and if phylogenetic inference indicates that a WGD is involved, the node is also labeled with an empty symbol. Additionally, tandem duplicates of *M. acuminata* are specified and labeled with the symbol #. Possible CHK pseudogenes from *M. acuminata* and *Z. mays* are given in italic. **b** Alternative tree topology within the AHK4 clade (monocots) of the reconciled maximum likelihood tree (codon substitution)

To analyze the ancient duplication events during cytokinin receptor evolution in more detail, the above-described set of gene trees (ML; Mr. Bayes, codon, protein, nucleotides trees) were reconciled with a species tree that reflected the commonly accepted evolution of land plants (Fig. 1). Furthermore, genomic organization concerning location in collinear or syntenic blocks was studied (Additional file 2: Table S2). The results are summarized in Table 2 and information on colinearity is included in the reconciled CHK tree (Fig. 2).

**Table 2:** Gene colinearity of the cytokinin signaling components

Gene pair	Multiplicon	Anchor points^a^	Ks/median Ks^b^
BrarapCHK04/CHK03	340,248	5	1.03/1.05
	Syntenic region		
ZeamayCHK03/CHK04	11,281	135	0.19/0.26
ZeamayCHK05/CHK06	10,125	315	0.15/0.29
GosraiCHK02/CHK06	59,516	31	0.39/0.48
GosraiCHK02/CHK04	65,400	27	0.34/0.52
GosraiCHK04/CHK06	36,049	78	0.38/0.60
GosraiCHK03/CHK07	36,049	78	0.47/0.60
GosraiCHK03/CHK08	125,670	13	0.47/0.69
GosraiCHK07/CHK08	148,631	11	0.43/0.46
GosraiCHK01/CHK05	98,488	17	0.39/0.57
MusacuCHK05/CHK15	11,287	133	0.37/0.54
BrarapHPT01/HPT03	52,943	36	1.12/1.24
BrarapHPT01/HPT02	296,202	6	0.93/1.34
BrarapHPT02/HPT03	45,950	44	0.32/0.38
BrarapHPT07/HPT08	29,877	334	0.63/0.37
ArathaHPT01/HPT02	99,381	17	0.50/1.19
MusacuHPT06/HPT09	17,637	17	0.36/0.66
MusacuHPT07/HPT09	39,756	6	0.36/0.85
ZeamayHPT01/HPT02	23,667	10	0.12/0.28
OrsaJaHPT01/HPT02	20,328	13	0.83/1.16
OrsaJaHPT03/HPT04	13,603	40	1.02/1.15
BrarapRRA02/RRA12	163,623	10	0.38/0.35
BrarapRRA02/RRA07	65,158	27	0.47/0.37
BrarapRRA07/RRA12	31,421	160	0.30/0.39
BrarapRRA07/RRA11	49,477	40	0.87/1.05
BrarapRRA04/RRA06	151,125	22	0.84/1.13
BrarapRRA06/RRA10	56,429	33	0.29/0.39
BrarapRRA08/RRA09	30,151	290	0.43/0.37
BrarapRRA01/RRA03	31,812	143	0.41/0.36
BrarapRRA17/RRA13	82,131	21	1.01/1.01
BrarapRRA17/RRA20	101,049	18	0.92/1.02
BrarapRRA17/RRA21	29,311	402	0.35/0.37
BrarapRRA21/RRA13	65,663	27	0.11/1.03
BrarapRRA21/RRA22	39,677	61	1.20/1.20
BrarapRRA13/RRA22	30,346	258	0.25/0.36
BrarapRRA13/RRA20	31,080	178	0.27/0.38
BrarapRRA14/RRA15	61,084	30	0.79/1.19
BrarapRRA14/RRA18	30,346	258	0.26/0.36
BrarapRRA15/RRA18	40,545	58	1.05/1.11
BrarapRRA15/RRA22	350,553	5	16.41/2.30
OrsaJaRRA06/RRA07	10,605	211	1.30/1.30
OrsaJaRRA08/RRA09	12,741	56	1.47/1.33
OrsaJaRRA01/RRA02	10,348	252	0.02/0.1
AralyrRRA02/RRA04	36,506	75	1.1/0.91
AralyrRRA05/RRA07	60,452	30	0.96/0.88
AralyrRRA01/RRA03	31,061	179	0.62/0.88
AralyrRRA09/RRA10	35,707	80	0.87/0.82
ArathaRRA03/RRA04	34,065	96	0.83/0.91
ArathaRRA05/RRA06	56,388	33	1.18/0.96
ArathaRRA03/RRA04	34,327	93	0.77/0.96
ArathaRRA07/RRA08	30,310	263	1.18/0.9
ArathaRRA09/RRA11	30,310	263	0.74/0.9
ZeamayRRA05/RRA08	11,007	162	1.26/0.26
ZeamayRRA10/RRA12	33,006	7	1.87/2.23
ZeamayRRA11/RRA12	11,559	116	0.40/0.33
MusacuRRA06/RRA07	37,422	6	0.98/0.66
MusacuRRA07/RRA08	35,224	7	1.45/1.16
MusacuRRA11/RRA14	16,973	19	0.50/0.50
MusacuRRA11/RRA13	35,273	7	0.73/0.64
MusacuRRA03/RRA20	15,306	25	0.74/0.60
MusacuRRA01/RRA02	25,508	9	0.41/0.41
MusacuRRA02/RRA04	16,323	21	0.53/0.72
BrarapRRB41/RRB42	33,554	102	0.33/0.34
BrarapRRB32/RRB33	30,151	290	0.36/0.37
BrarapRRB37/RRB39	40,489	58	0.48/0.42
BrarapRRB47/RRB48	122,822	14	0.38/0.33
BrarapRRB59/RRB60	44,640	47	0.56/0.39
AralyrRRB18/RRB16	70,367	25	1.07/0.92
AralyrRRB25/RRB24	105,708	16	0.93/0.78
AralyrRRB37/RRB25	234,790	7	6.90/6.90
ArathaRRB22/RRB19	56,277	33	1.31/0.82
ArathaRRB23/RRB24	111,168	15	0.95/1.19
ArathaRRB23/RRB45	268,141	6	8.89/4.45
OrsaJaRRB21/RRB27	12,291	73	1.34/1.34
OrsaJaRRB21/RRB27	13,539	41	0.98/1.10
ZeamayRRB24/RRB25	14,587	30	1.30/1.79
ZeamayRRB21/RRB23	10,224	280	0.12/0.25
ZeamayRRB18/RRB27	10,125	315	0.14/0.29
MusacuRRB30/RRB33	17,165	18	0.40/0.56
MusacuRRB30/RRB36	17,906	17	0.49/0.55
MusacuRRB39/RRB42	16,390	21	0.47/0.72
MusacuRRB53/RRB57	17,546	17	0.53/0.53

^a^colinear gene pairs

^b^Ks distance of the gene pair given in column 1/median Ks distance of the multiplicon

All reconciled trees supported two ancient duplication events: *i*) one duplication before the split of gymnosperm and angiosperms giving rise to the ancestor of the AHK4 clade and the ancestor of the AHK2/AHK3 clade (Fig. 2, Additional file 3: Figure S3) which coincides with the ancient ζ WGD event [37]; *ii*) a second duplication event before the radiation of angiosperms giving rise to the AHK2 and AHK3 clades which coincides with a well-established ε WGD event at the basis of angiosperm evolution [37]. A third ancient duplication event is predicted within the monocot clade of AHK4. The different reconciled trees support either a correlation with the described commelinid specific τ WGD [40] (Fig. 2a) or the grass specific σ WGD [41] (Fig. 2b). Lineage specific duplications of CHKs are supported by the reconciled trees in *Z. mays, Gossypium raimondii*, and *M. acuminata* that correlate with lineage specific WGDs. Exactely these species all have a CHK copy number greater than four (see Table 1).

While genomic comparisons did not provide further evidence that the above-described ancient duplications could be traced back to the discussed WGD events, these comparisons could clearly show that the lineage specific WGD in *Z. mays* and *G. raimondii* were involved in the copy number increase in these species. For example, in *Z. mays*, the paralogous CHK pairs (ZeamayCHK01/CHK02, ZeamayCHK03/CHK04, ZeamayCHK05/CHK06) are located in collinear or syntenic regions (Fig. 2; Table 2; Additional file 2: Table S2). Furthermore, the estimated divergence time of the collinear regions (i.e., the paralogous blocks) based on pairwise synonymous nucleotide substitution rates (Ks distances) vary between 0.15 and 0.29 and correlate well with the Ks distance characteristic for gene pairs created by the maize-lineage specific WGD [42, 43]. Interestingly, only three of the four duplicates resulting from this WGD were retained (Fig. 2). In *G. raimondii,* genomic organization and Ks distances also supports the lineage-specific gene-expansion of CHKs via the five- to six-fold ploidy increase over ~ 60 Mya (Fig. 2; Table 2), characterized by Ks distances ~ 0.5 [44]. In *M. acuminata* however, which possesses eight CHK-encoding gene copies and experienced three rounds of WGD in the range of 65 to 100 Mya [21], intragenomic comparisons only support an origin of the paralogs MusacuCHK15/CHK5 (Table 2) through one of these events. Besides, one pair of tandem duplicates, MusacuCHK01/CHK03, has been identified within an ultracontig of the *M. acuminata* genome. One further duplication event, which led to a copy number increase in *Brassica* sp., could be identified as a large scale duplication. The paralogs BrarapCHK04/CHK03 are located within an collinear intragenomic region (Fig. 2, Additional file 2: Table S2). Based on phylogenetic reconstruction and Ks distances, they originated from the Brassicaceae-wide α WGD.

Concerning the functionality of the above mentioned additional CHK copies in *Z. mays*, *G. raimondii* and *M. acuminata*, in silico analyses of transmembrane helices predict only three of the eight MusacuCHK copies (MusacuCHK01, CHK06 andCHK15) and five of the seven ZeamayCHK copies (ZeamayCHK03–07) encode functional receptors while all CHKs from *G. raimondii* are predicted to be functional. Additionally, for ZeamayCHK01/CHK02/CHK04 secretory signal peptides that targets its passenger protein for translocation across the endoplasmic reticulum membrane in eukaryotes are predicted.

In summary, given the scenario of ancient origin of three CHK copies together with the repeated WGDs that occurred in the subsequent radiation of angiosperm species, this indicates that the most common fate of CHK duplicates resulting from WGDs is gene loss. Only in some species that experienced rather recent polyploidization events (Ks values 0.1–0.4) have several duplicates been retained.

### HPT copy number increased through palaeo- and mesopolyploidy events

We analyzed and reconstructed the evolution of HPT-encoding genes analogous to the CHKs by combining phylogenetic tree reconstruction, gene tree-species tree reconciliation, and gene synteny analyses to obtain predictions of duplication events and their timing. Compared with the analyses of CHKs, a reduced dataset including only angiosperm species was used as the alignable region of the HPTs covering only the 85 amino acids of the PFAM HPT domain contained limited phylogenetic signal. The HPT copy number in the analyzed species ranged from five to 10 (Table 1).

**Fig. 3:**
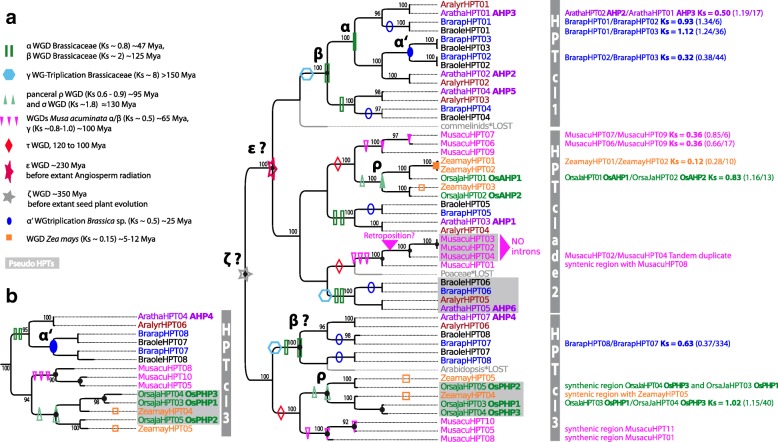
Reconciled tree of HPT encoding sequences. Pseudo HPTs without the canonical histidine are marked with a gray box. **a** Reconciled Bayesian HPT tree (codon substitution model). Figure style is analogous to that of Fig. 2 concerning color code and the labeling of duplication events. Gene tree reconciliation includes the rearrangement of branches with a support less than 90% (posterior probability). Branch support of > 90% is given in the tree. The three dominant clades of HPTs and tandem duplicates are indicated. **b** Alternative maximum likelihood tree topology of HPT clade 3, supporting the proposal that the gene pair BrarapHPT8/HPT7 originated by the α’ WGT

The reconciled trees supported two different topologies with either two or three repeated duplications before the split of mono- and dicots (Fig. 3a and Additional file 3: Figure S4) indicating that *ζ* and *ε* WGD may have been involved in ancestral HPT amplification. According to the reconciled tree, HPTs experienced further lineage-specific amplification in mono- and dicots through WGDs. For example, three of the five HPTs from *A. thaliana* (AHP2, AHP3 and AHP5) are found in a clade specific for dicots (HPT clade 1, Fig. 3a) and most likely originated by the *Brassicaceae-*specific α and β WGD (Fig. 3a, Additional file 2: Table S2). Gene colinearity of AHP2 and AHP3 support this phylogeny based reconstruction of WGD events (Table 2, Fig. 2). In contrast, the other two HPTs from *A. thaliana* that group with monocot HPTs were not amplified by the *Brassicaceae-*specific WGDs, meaning that the resulting duplicates have been lost (Fig. 3a, HPT clade 2 and 3). The monocot HPTs increased either via the pancereal σ or ρ WGD (Fig. 3a, HPT clade 2) which is supported by colinearity of OsAHP1/OsAHP2 and OsPHP1/OsPHP3. However, with the limitations of Ks distance based dating in mind, it is not possible to clearly identify which of the two WGD was involved (Table 2, Fig. 3). The duplicates resulting from the mesopolyploidy events in the lineage towards *Brassica* and *Z. mays* were predominantly lost, except the paralogous pairs BrarapHPT02/HPT03, BrarapHPT07/HPT08, and ZeamayHPT01/HPT02 (Fig. 3b). All three pairs are located in colinear regions with Ks distances similar to the duplicates of the lineage specific polyploidy events. HPTs in *M. acuminata* showed similar to CHKs a highly species-specific evolutionary pattern and included tandem duplications and/or possibly retroposition as evidenced by the observation that MusacuHPT02/HPT04 are located on one chromosome in close vicinity (chromosome 7) and MusacuHPT02/HPT03/HPT04 completely lack introns.

Concerning the evolution of pseudo-HPTs and thus the evolution of a new regulatory component in cytokinin signaling, the phylogenetic reconstructions support that they have emerged at least twice independently. The duplicability of monocot pseudo-HPTs (OsPHP1/OsPHP3, see above) differed from the dicot pseudo-HPTs as the former were amplified via a lineage-specific WGD event (*ρ* and/or *σ*) while the latter were not amplified.

In summary, the most dominant fate of HPT duplicates that arose from WGD events was again gene loss. However, some HPTs copies were retained after the *β*, *α*, *α*,*’* and *ρ* WGD events which in comparison did not led to a similar copy number increase in the upstream acting CHKs.

### Response regulator families expand continuously via repeated WGD

The evolution and duplication pattern of RRAs and RRBs of mono- and dicots was reconstructed separately by using the same approach as that for CHKs and HPTs. The 112-amino-acid PFAM response regulator receiver (RR) domain was used as alignable region. However, the tree signal of RRBs was poor indicated by a relative high percentage of low branch supports. Thus, the threshold for the branch support for gene tree reconciliation in the RRB trees was lowered to > 0.75 for SH-like branch support. Furthermore, RRB trees reconstructed with maximum likelihood and Bayesian inference were incongruent with regard to the basal branching pattern and also the placing of some highly diverged RRB sequences from *M. acuminata* (MusacuRRB38, RRB40, RRB43), but individual clades were consistent between the two reconstructions (Additional file 3: Figure S5 and S6). These branches are highlighted in Fig. 5.

**Fig. 4:**
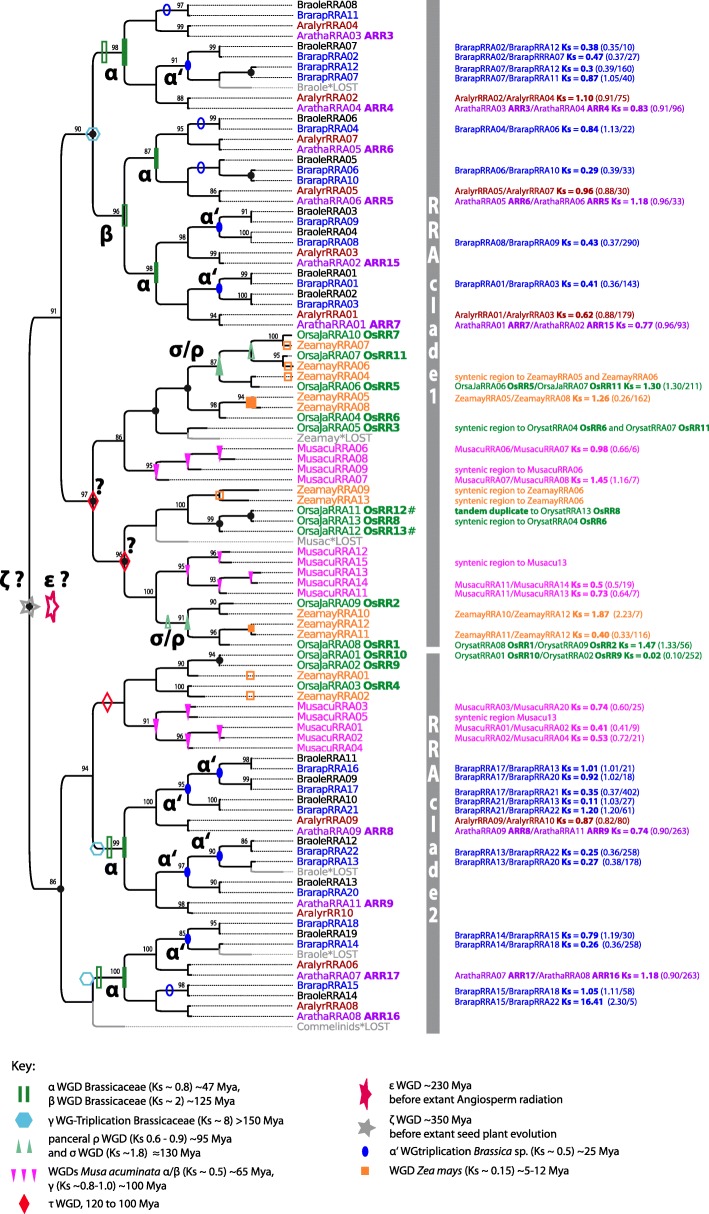
Reconciled maximum likelihood tree of RRA encoding sequences. Figure style is analogous to that in Fig. 2 concerning color code and the labeling of duplication events. Gene tree reconciliation includes the rearrangement of branches with a support less than 85% (SH-like branch support). Branch support of > 85% is given in the tree. The two dominant clades of RRAs are indicated

Although similar numbers of RRAs and RRBs exist in flowering plants (Table 1), the in-depth phylogenetic analyses show that their evolutionary pattern is different. For RRAs, the reconciled trees support a scenario of constant copy number increase via repeated paleao- and mesopolyploidy events. The RRAs of monocots and dicots form two main groups indicating that their last common ancestor possessed two RRA copies, which might have arisen from the ancient ζ or ε WGD. Duplication and differentiation of RRAs then occurred independently during monocot and dicot radiation leading to the observed two main clades and the increase in copy number in both plant groups. For example, the phylogenetic analyses indicate that *β* and *γ* WGD were involved in the expansion of RRAs within clade 1 (Fig. 4). Phylogeny and collinearity suggest an origin of four paralogous RRA pairs in *Arabidopsis thaliana* (ARR6/ARR5, ARR15/ARR7, ARR8/ARR9, ARR17/ARR16) via the *α* WGD event. Thus, RRA duplicates were more likely to be retained compared to the CHKs and HPTs evolution.

**Fig. 5:**
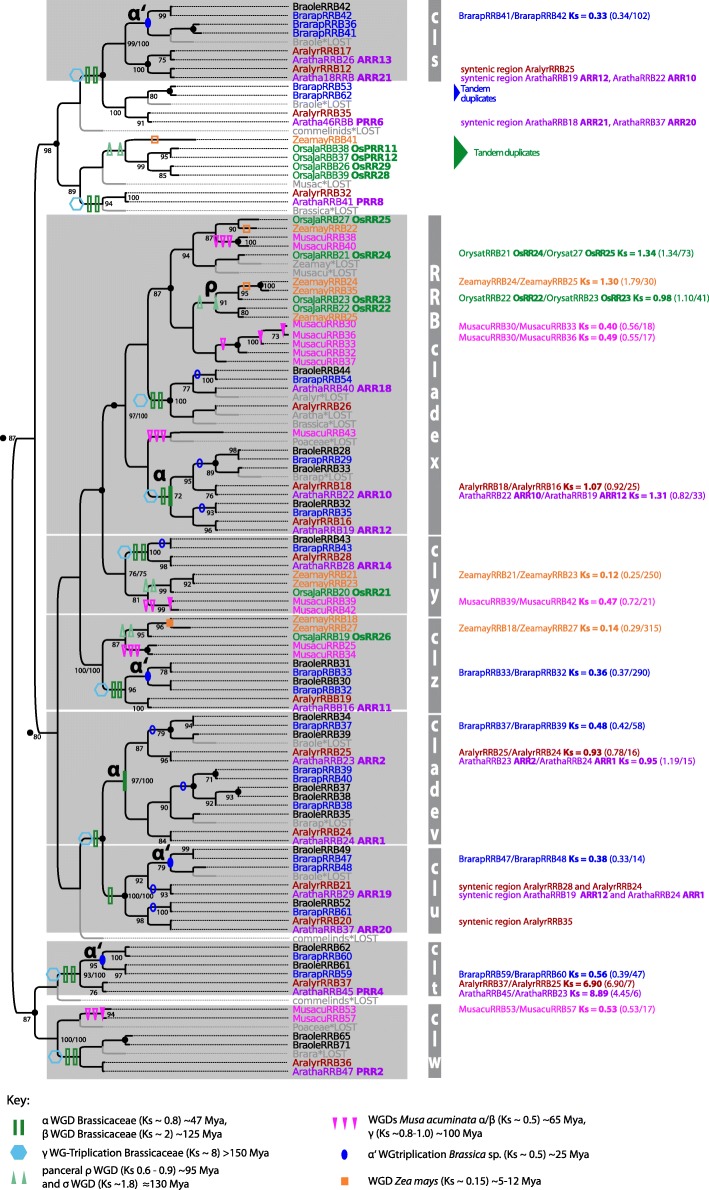
Reconciled maximum likelihood tree of RRB encoding sequences. Figure style is analogous to that of Fig. 2 concerning color code and the labeling of duplication events. Gene tree reconciliation includes the rearrangement of branches with a support less than 70% (SH-like branch support). Branching pattern in Bayesian RRB tree reconstructions differed for basal branches from maximum likelihood tree reconstructions. Groups that are consistently reconstructed in Bayesian and maximum likelihood trees are marked with gray boxes. Branch support of > 70% (SH-like branch support/posterior probability) is given in the tree

On the other hand, RRBs show a more heterogeneous pattern of evolution. Some clades share sequences from monocots and dicots (Fig. 5, clades w - z), suggesting that the last common ancestor possessed at least four RRB copies. Of note, within three of these clades, which include *A. thaliana* PRR2, ARR11, and ARR14 (clade w, y, z), WGD events rarely led to an increase of the copy number. However, individual RRB copies, such as the *A. thaliana* ARR10/ARR12 and ARR2/ARR1 gene pairs, originated via WGD events. Compared with RRAs, amplification of RRBs via WGD is patchier. Moreover, RRBs show one more noticeable feature: some gene copies in *O. sativa subspec. Japonica* [45] and *B. rapa* originated from tandem duplication.

To summarize, RRAs and RRBs differ in their evolutionary pattern, although both gene families show a high gene duplicability. RRAs exhibit a clear trend towards gene retention instead of gene loss after WGD. RRBs may have been amplified by additional duplication mechanisms, e.g., tandem duplication. However, the reconstruction of RRBs evolution is in general vaguer, suggesting that they might comprise a functionally heterogeneous group of sequences.

## Discussion

### Genes encoding cytokinin receptors were recruited early in land plant evolution for cytokinin signaling

The results of our phylogenetic reconstructions of CHK, HPT, RRA, and RRB evolution are in solid agreement with similar, albeit smaller-scale studies [25, 26, 45, 46] and support an early origin of cytokinin signaling at the base of land plant evolution, and functional cytokinin signaling perhaps is even present in *K. flaccidum*. However, the CHKs of *K. flaccidum,* which form a monophyletic clade at the basis of the CHK gene trees, group next to a group of CHKs from the early diverging land plant *P. patens* recently identified by Gruhn et al. [2]. Although a heterologously expressed member of this subfamily (PpCHK4) has previously been shown to be able to bind cytokinin hormones and to translate this binding into a cellular response in a dose-dependent manner [46], in *P. patens* a group of three further CHKs evolved which clade with the classic cytokinin receptors from the other land plants [46]. Genetic and biochemical experiments have shown that these three classic cytokinin receptors are necessary to mediate the cytokinin response in this moss [27]. One can speculate at this point that these three copies were recruited to function specifically in cytokinin signaling and have evolved from ancestors forming the very basal group of CHKs from *K. flaccidum* and *P. patens*, whose biological function needs to be investigated.

Interestingly, HPTs and RRBs are also present in Chlorophyceae algae, the branch of the green algae that did not give rise to land plants [47]. However, their function in these green algae is unclear. Thus, we are tempted to speculate that the CHKs might have been recruited specifically in the Charophyceae lineage to serve as a new “plug-in” for the established HPT-RRB network. As has recently been proposed, the reorganization of gene regulatory network architecture is a major factor underlying evolution, and phytohormonal pathways might be used to redirect such gene regulatory networks to fulfill new functions [48].

### Cytokinin signaling pathway expanded via WGD events

The applied approach of integrating phylogenetic analyses with comparisons of genome collinearity, which has previously been successfully applied to identify ancient WGD in monocots [40], indicates that many of the duplications that have affected cytokinin signaling components are genome-wide or other large-scale duplications. Also for ethylene signaling in *M. acuminata,* WGD has been shown to be the dominant duplication mode [49]. However, a few exceptions can be found to this common trend. In *O. sativum subspec. Japonica*, the RRBs OsPRR11, OsPRR12, OsRR29, and OsRR28 originated by tandem duplications (Fig. 5), consistent with repeated unequal cross-overs [45]. In *M. acuminata*, the genes encoding MusacuHPT02/HPT04 have been identified as tandem duplicates. Both copies are intronless. MusacuHPT03 is closely related to this pair of HPTs and also lacks introns. One can speculate that retroposition was involved in the origin of these three copies; this would represent yet another mechanism for gene duplication affecting members of the cytokinin signaling pathway.

In order to estimate the timing of WGD events that affected cytokinin signaling, we have used Ks distances between paralogous pairs located in collinear intragenomic blocks. These distances only offer rough dates that have to be taken with caution because of variable rates among the different gene families [40] and between species [50, 51]. Nevertheless, the WGDs identified as affecting cytokinin signaling by using this approach are in good agreement with those in previous studies. A genome alignment spanning major Poaceae lineages supports the amplification of the cytokinin signaling components through WGD events in this lineage [51]. Furthermore, a phylogenomic analysis of ancestral polyploidy events indicates that RRAs and CHKs were amplified before the split of basal angiosperms (*Aristolochia*, *Liriodendron*, *Nuphar*, and *Amborella*), most likely via the *ε* WGD [37].

### Patterns of GDB in cytokinin signaling

**Fig. 6:**
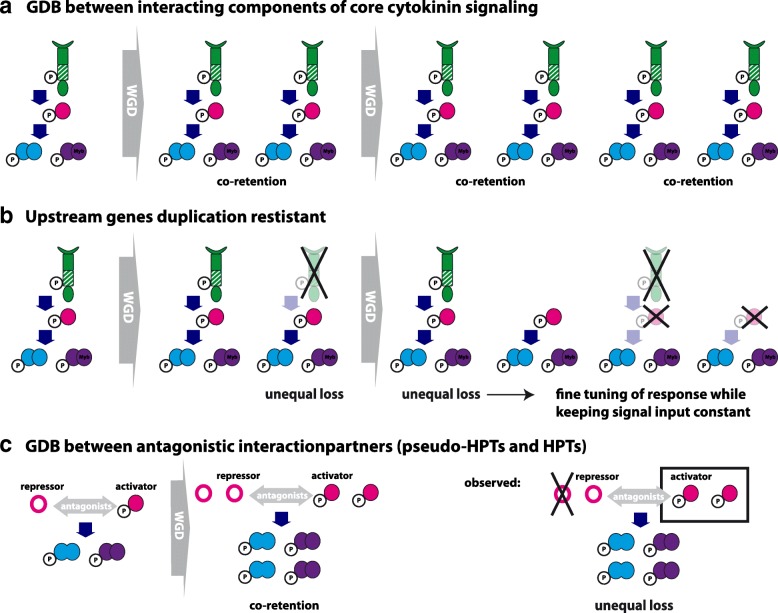
Schematic illustration of the discussed fate of core cytokinin signaling after WGD events. **a** Gene dosage balance (GDB) between interacting components of core cytokinin signaling predicts co-retention of the encoding genes after WGD leading to a concerted increase of upstream and downstream elements. **b** Upstream genes, i.e. CHKs that mediate signal input, are duplication resistant compared to downstream element leading to a unequal increase of the individual components of the signaling pathway. In this scenario, perceiving of the signal is conserved but the output can be individually regulated by diverse regulators. **c** GDB between antagonistic HPTs and pseudo-HPT predicts co-retention. Unequal loss could result in a shift towards either the activator or repressor, depending on which gene is lost

Of special interest in this study has been the testing of whether the cytokinin signaling components show a signature of GDB. Many previous studies have implied that GDB preferentially “acts” on multiprotein complexes and signal transduction/regulatory networks [15, 17, 20]. The central assumption is: if a pathway, whose components are linked in a dosage-sensitive relationship, is duplicated as a whole via a WGD event, the relative dosage between genes will be preserved, and the duplicated dosage-sensitive genes will be preferentially retained. Thus, “interacting” genes are bound to be co-retained over evolutionary time [15]. In core cytokinin signaling, interacting genes are CHKs, HPTs, RRAs and RRBs (Fig. 1a) and if GDB is a dominating force, co-retention after WGD of these components is predicted (Fig. 6a), leading to a concerted increase of all interacting genes. According to our data, however, a different trend exists in that downstream elements in cytokinin signaling are more likely to be retained while the upstream elements tend to be lost (Fig. 6b). This pattern is in agreement to the trend that upstream genes in a biochemical pathway evolve more slowly than downstream genes [52, 53], which might be explained by network characteristics, as most likely the rate- or flux-controlling elements on which natural selection preferentially acts are the upstream elements in a pathway [54].

Focusing on the upstream part – the CHKs, which channel the signal into cytokinin signaling – we found relatively simple orthologous relationships, and flowering plants have a similar repertoire of CHKs [45, 46]. Whereas CHKs were amplified before the split of angiosperms possibly via WGD events, the subsequent repeated rounds of palaeopolyploidy events during flowering plant evolution did not affect copy number of CHKs, meaning that the resulting duplicates were lost. This pattern of an unique amplification at the trunk of a tree with very few or no subsequent additions of gene copies is consistent with the phenomenon of “frozen duplications” described by Makarova et al. [55] as evolutionarily stable paralogous clusters that are not further amplified. Only the more recent mesopolyploidy events led to a copy number increase of CHKs in *G. raimondii, Z. mays*, and possibly also in *M. acuminata*. In the two latter species however, several of the additional copies contain extra sequence motifs like transmembrane helices or secretory signal peptides possibly rendering them non-functional in cytokinin signaling. One can speculate, that these copies are in the process of pseudogenization and eventually will be purged from the genome, which may also be the long-term fate for the additional CHK copies in *G. raimondii.*

HPTs, RRAs, and RRBs displayed overall a higher degree of lineage-specific expansion compared with that of CHKs. However, the in-depth phylogenetic analyses showed that the copy number increase of these components can be traced back to different polyploidy events meaning that the downstream parts were not amplified in a concerted way. Especially striking is the different evolutionary pattern of RRAs and RRBs. While RRAs show a continuous increase in copy number via WGDs, RRBs are much more heterogenous. But, RRBs comprise in general a more heterogenous group of genes. Most but not all of the RRB genes encode an additional Myb domain and some RRBs lack the conserved phosphorylation site. Functional differentiation between these classes is not well understood and thus, the present analyses might guide further functional studies.

Another interesting aspect of the cytokinin signaling are the pseudo-HPTs. GDB is especially suggested to be a driving force between proteins with opposing actions, such as enzymatic or transcriptional activators and inhibitors within a pathway, and might shape the duplicability of the encoding genes [15]. Within the cytokinin signaling pathway the HPTs and the pseudo-HPTs, which lack the conserved His residue, are discussed to be antagonistic players and thus predicted to be co-retained after WGD (Fig. 6c). Of the six HPTs from *A. thaliana*, only AHP6 is a pseudo-HPT [24, 32]. It negatively interferes with pathway activity, most likely by competing with AHP1–5 for interaction with the phosphorelay machinery [31, 56]. Whereas in *A. thaliana* only one pseudo-HPT (AHP6) has been identified, this group is especially large in rice with three copies (OsPHP1, PHP2, and PHP3) (Fig. 3). Regarding duplicability, or co-retention after WGD, pseudo-HPTs strongly differ from HPTs. For example, the ancestor of AHP6 and its orthologs in *Brassica* experienced according to the reconstructed phylogeny up to three WGD events (α, β, γ) but the resulting duplicates have not been retained (Fig. 3). However, the corresponding antagonists (AHP2, AHP3, AHP5) were partly amplified via the α and β WGDs. Thus, in *Arabidopsis* and *Brassica*, either the pseudo-HPTs experienced unequal loss after WGDs (Fig. 6c), or their function as repressor evolved only after the last common WGD. In rice, both the pseudo- and the authentic-HPT gene family show a copy number increase due to polyploidy events, however most likely different, pancereal WGD events were involved. Thus, no pattern of co-retention between pseudo-HPT and authentic HPTs is observed.

In general, lineage-specific gene family expansions seem to be one of the principal means of adaptation and one of the most important sources of organizational and regulatory diversity in crown-group eukaryotes [57]. In cytokinin signaling, expansion is mostly found for downstream elements. This amplification might allow the regulatory fine tuning (subfunctionalization) or the rewiring of signal output to execute novel functionality. Furthermore, no unique pattern of co-retention after WGDs, indicative of GDB among the encoded proteins, was identified in cytokinin signaling. Possible explanations for this result are: *i)* an important prerequisite for GDB to manifest is that the duplicates are co-expressed. While immediately after a WGD event paralogs show an identical expression pattern as coding as well as regulatory sequences have been duplicated, expression pattern subsequently can change. Indeed, expression divergence between paralogs seems to be the rule rather than the exception [58] and represents an important way of subfunctionalization between duplicates. Furthermore, gene dosage balance can be compensated by regulatory changes [18]. To further test the potential role of GDB in the evolution of cytokinin signaling, detailed co-expression studies of the interacting components (Fig. 6a), the antagonistic interactions partners (Fig. 6c) as well as their duplicated copies in polyploids of different age are necessary. GDB predicts that interaction partners should show a correlated expression level. Thus, after a WGD, an unequal loss of one of the interactions partners needs to be compensated according to the GDB. For several model plants, online tools to study expression pattern are available to get first insights in the dynamic of duplication and expression pattern, e.g. the Bio-Analytic Resource for Plant Biology (bar.utoronto.ca). And *ii*) a central assumption of GDB, namely that gene expression is directly correlated to copy number variation (CNV), may be not valid. For example, CNV in humans only partly account for the differences in expression between individuals, whereas a large portion of the variance must stem from other sources [35]. Furthermore, most genes present in CNVs in *Drosophila melangolaster* show no evidence of increased or diminished transcription [59]. Thus, this central assumption of GDB needs to be further studied.

However, while GDB may be of minor importance to co-stabilize duplicates of cytokinin signaling in the long run, in *M. acuminata*, two antagonistic components of ethylene signaling have been shown to be co-retained after the three lineage-specific WGD events indicating that GDB shaped their evolution [49]. Together, these results reflect the complexity of molecular mechanisms that shape gene duplicability. The fixation and the retention of duplicated genes in plant genomes seem to be context-dependent events, and relevant factors include intrinsic properties, such as gene function, and the environment in which the duplication occurred [60, 61]. Several specific gene features including gene ontology (GO-slim classification), sequence-related features (gene and protein sizes, the GC content in the third codon position, protein domain size), expression-related features (level of expression and biotic and abiotic responsiveness), and conservation-related features (omega, the ratio of relative fixation rates of synonymous and nonsynonymous mutations, as an indicator of selective pressure), have been shown to influence gene retention after WGD [56]. Another important feature of proteins effecting the duplicability of the encoding genes might be their tendency to form symmetrical homomers. Duplication of a gene that encodes a homomeric protein can lead to the phenomenon of paralog interference, which basically predicts a functional link between paralogous genes via interactions of the encoded proteins in multimeric complexes and one important outcome of paralog interference is a prolonged retention timer after duplication [62]. Examples of paralog interference has been described for MADS box transcriptional regulators in the yeast *Kluyveromyces lactis* [63] and steroid receptors [64], both form dimers. Of note, also cytokinin receptors are also thought to function as dimers [28, 65] and paralog interference could influence their duplicability. A prolonged retention of CHKs in *Z. mays* compared to HPTs, RRAs and RRBs (compare Figs. 2, 3, 4 and 5) could be an indicator but further studies of protein interactions are necessary.

## Conclusions

The observation of the non-random loss of genes following WGD has stimulated much discussion regarding the molecular mechanisms that influence these outcomes. The pattern of gene retention after WGDs within the cytokinin signaling pathway fits best to the model stating that downstream genes in a pathway evolve faster. However, most striking is the heterogeneous pattern of gene retention of the various components of cytokinin signaling and the diverse modes of duplication as besides WGD also tandem duplication and possibly retroposition was found. Obviously, various mechanisms at diverse levels of interaction act in shaping the evolution of this signal transduction pathway, all of which require further experimental exploration. Detailed mechanistic studies of specific candidate genes, e.g., young paralogs in neopolyploid species, including analyses of gene expression, gene function (in vivo and in vitro), and protein interactions will allow to gain a more complete picture of the forces that shape the fate of a duplicated gene.

## Methods

### Identification of cytokinin receptor encoding sequences

Sequences encoding putative or confirmed cytokinin receptors were obtained from the genomes and transcriptomes of selected species (Additional file 1: Table S1, Additional file 4: alignment 1) by screening the online platforms “Phytozome”, “Ensemble”, and “1 K database” [66–68] by using the Basic Local Alignment Search Tool (BLAST). Species were chosen to cover the evolution of cytokinin signaling during land plant evolution by using the charophyte alga *Klebsormidium flaccidum* [25] as an outgroup. For each species, we performed an iterative BLASTN search with the sequences encoding the Cyclase Histidine kinase Associated Sensory Extracellular (CHASE) domain of cytokinin receptors of the model plant *Arabidopsis thaliana* as query sequences (NCBI accession numbers AHK4 NM_201667, AHK2 BT002530, AHK3 NM_102494)*.* The CHASE domain is the ligand binding domain of cytokinin receptors [29]. Subsequently we used every identified sequence again as query to search for further sequences with homology to cytokinin receptors in the respective species. Sequences were named as *CHASE-domain containing His kinase* (CHK) according to the common nomenclature [69] and numbered serially. For the simple and rapid identification of the species, the first three initials of the genus and of the species name are given, e.g., Aratha for *Arabidopsis thaliana* (Additional file 1: Table S1). To differentiate between *Oryza sativa ssp. japonia* and *Oryza sativa ssp. indica*, the first two initials of the genus, species, and subspecies name are given. Furthermore, we added the annotation of well-characterized cytokinin receptors from *Arabidopsis thaliana* [70] and *Oryza sativa ssp. japonica* [71]. If different splicing variants of a gene were identified, only the longest variant was used for subsequent analyses. The individual sequences of this sequence collection were analyzed regarding their protein domain structure by using the PFAM database [72]. Sequences that comprised the following four PFAM protein domains were identified as functional cytokinin receptors: *i*) CHASE domain (PF03924); *ii*) Response regulator receiver domain (PF00072); *iii*) Histidine kinase-, DNA gyrase B-, HSP90-like ATPase domain (PF02518); and *iv*) His Kinase A (phospho-acceptor) domain (PF00512). As we are interested in the retention of functional genes after WGDs, stringent selection criteria were applied, and sequences that lacked more than 50% in one of the four domains were rejected. The resulting dataset of 166 sequences was used for phylogenetic analyses. Genes encoding cytokinin receptors were also analyzed concerning the presence of transmembrane helices with the “TMHMM Server v. 2.0” [73] and the presence of signal peptides with the SignalP [74] and Phobius [75].

### Identification of HPT and RR genes

To investigate the evolutionary pattern of the whole cytokinin signaling pathway, we additionally identified HPT and RR encoding genes in a subset of species that covered flowering plants (Additional file 4: alignment 2–4). We followed the same strategy as that described above, performing an iterative BLASTN search starting with sequences encoding HPT and RR domains from *Arabidopsis thaliana* [69]. We also used the PFAM database [72] to analyze the protein domain structure of the identified sequences. Sequences that lacked more than 15% of RR (PF00072)- and HPT (PF01627)-PFAM domains were rejected. For phylogenetic analysis, only the conserved regions encoding the HPT and RR PFAM domain were used as the alignable region. HPT encoding sequences were further classified as authentic His-containing phosphotransfer proteins if they contained a conserved histidine residue, and sequences that lacked this site were classified as pseudo His-containing phosphotransfer proteins [76]. Moreover, response regulators were further categorized into type-A, type-B, type-C, and clock related- and pseudo-response regulators and named as RRA, RRB, RRC, and PRR, respectively [69]. We assigned the sequences of our dataset to these categories based on phylogenetic analysis as previously suggested [69]. We used the well-characterized set of RRs from *A. thaliana* as a reference (see [38]) in these phylogenetic analyses and compared the individual species-specific sets of RRs with this reference. For the more divergent species investigated in this study (*P. abies, P taeda, S. moellendorffii*, and *K. flaccidum*), *A trichopoda* was additionally included in the comparative phylogenetic analyses to classify RRs. To perform these analyses, we calculated multiple sequence alignments with the MUSCLE algorithm [77] implemented in MEGA Software package, version 6.06 [78] and calculated Maximum Likelihood trees with MEGA based on the General Time Reversible (GTR) substitution model with a Gamma distribution to model evolutionary rate differences among sites (5 categories) with 100 bootstrap replicates. Sites with less than 95% site coverage were excluded from the analyses. The RRAs were clearly distinguished as they formed a well-supported monophlyetic clade, even in phylogenetic analysis with diverse species. The same is true for RRCs, which clearly group with the response regulator receiver domain from cytokinin receptors [46]. The PRRs were identified by their phylogenetic position and by an additional characteristic protein motif: the Constans/Constans-like/TOC1 (CCT) domain [46]. The remaining response regulators were designated as RRBs. This group is heterogeneous concerning its phylogenetic composition and includes response regulators that have an additional myeloblastosis (Myb)-related DNA binding domain [46] but also response regulators that lack this domain as well as response regulators that have been annotated as pseudo-response regulators by Suzuki et al. [79] based on alterations in the conserved phosphorylation site (the DDK motif) [80]. The biological roles played by latter, noncanonical members are not clear but based on the agreed nomenclature for cytokinin signaling components, they were included in the RRB group in this study [69]. When experimental evidence was available, the information was included in the analysis. Thus, based on work by Tsai and colleagues [45], OsRR27, OsRR31, OsRR32, OsRR30, and OsRR33 were shown not to function in cytokinin signaling, and thus, these sequences were excluded from our analyses. Furthermore, sequences that contained an additional EHD1 domain were excluded.

### Phylogenetic analyses

To reconstruct the process and pattern of evolution of the cytokinin signaling pathway during land plant evolution with emphasis on WGD events, the following datasets were assembled from the sequence collection for phylogenetic analyses: *i*) sequences encoding cytokinin receptors of land plants ranging from *Klebsormidium flaccidum* to monocots and dicots, and from monocots and dicots: *ii*) sequences encoding HPTs, *iii*) sequences encoding RRAs, and *iv*) sequences encoding RRBs. The respective sequences were aligned with the MAFFT multiple sequence alignment algorithm (MSA) [79] based on codons by using the GUIDANCE web server [81], which evaluates the confidence of the MSA. Based on the GUIDANCE confidence scores, unreliable columns were removed (threshold 0.93). We used ModelOMatic [39], which allows comparisons of nucleotide, amino acid, and codon models, to identify the best substitution model for subsequent tree reconstruction. For reconstructing the evolution of cytokinin receptors, the robustness of the reconstructed tree topology was tested by comparing phylogenetic trees based on nucleotide and codon substitution models. When reconstructing the evolution of HPTs, RRAs, and RRBs we focused on the best substitution model predicted by ModelOMatic. In general, maximum likelihood trees based on the general time reversible (GTR) nucleotide substitution model were calculated with RaxML [82], and the CAT approximation of rate heterogeneity was used to model rate heterogeneity. RaxML involves a rapid hill climbing algorithm to search for the best tree, with the subtree pruning re-grafting (SPR) starting on a randomized maximum parsimony tree. The initial rearrangement settings of SPR and the number of rate categories for the CAT approximation were optimized through comparative analysis by using various settings. Based on the original alignment, 20 tree inferences with the optimized settings were performed to find the best maximum likelihood tree according to the final likelihoods. Branch support was determined by 100 bootstrap repeats and mapped onto the best tree of the 20 inferences.

To calculate maximum likelihood trees under codon models, we used CodonPhyML [83]. We compared three codon models, namely *i*) the Goldman and Yang model [84], *ii*) the Muse and Gaut model [85], and *iii*) the YAP model [86], all of which differ in their instantaneous substitution rates between codons. The stationary frequency of codons and the transition-transversion ratio were estimated by maximum likelihood. The ratio of synonymous and nonsynonymous substitution rates was modeled as being constant over sites (M0 model). Site-rate variation was drawn from a discrete gamma distribution with four classes. Starting from an initial tree built by using the BioNJ algorithm, nearest neighbor interchange was used to search for the best tree topology. Branch support was assessed by using SH-aLRT statistics, which is a conservative test for branch support, comparable to standard bootstrap [87]. The tree with the highest log likelihood was selected for further analyses. In addition to the maximum likelihood approach, we used Bayesian inference to reconstruct phylogenetic trees (Mr. Bayes version 3.2 software; [88]). The following substitution models were used: *i*) the GTR model of DNA substitution with among-site variation drawn from a gamma distribution, *ii*) the Jones fixed-rate model of amino acid substitution, which was chosen via the model jumping option in Mr. Bayes, *iii*) the implemented codon model, which is based on the GY and MG codon models, to reconstruct phylogenetic trees. In the case of the codon model, the ratio of synonymous and nonsynonymous substitution rates (ω) was again modeled as being constant over sites. The posterior probabilities of the phylogenetic tree model were estimated as part of the Bayesian analyses by using Markov chain Monte Carlo sampling with Metropolis coupling running four chains in two simultaneous analyses. The analyses were run with uniform prior distributions for tree topology. A flat Dirichlet distribution (1.0) was used for stationary frequencies of nucleotides, codons, and amino acids, nucleotide substitution rates, and ω. Exponential priors were used for the shape parameter of the gamma distribution of rate variation. Branch lengths were unconstrained. The MCMC chain was sampled every 500 generations with the burn-in set to 25% from the cold chain. Convergence diagnostics were calculated every 5000 generations and analyses were continued until the average standard deviation of split frequencies reached 0.01. For all trees, cutoffs for the branch support were selected according to the tree signal, also for the subsequent gene tree reconciliation. Trees with a high percentage of low support were interpreted to contain low phylogenetic signal and to be less robust. To compare the tree topologies calculated based on different substitution models and the different algorithms (Mr. Bayes and maximum likelihood), we determined Robinson-Foulds distances (RF distance) [89] in R, package phangorn 2.4.0, and normalized them by dividing by the maximal possible distance [90]. RF-distance matrix is provided as Additional file 5: Table S3. For illustration, reconstructed RRB cladograms based on maximum likelihood (codonphyML, codon substitution model YAP CF3x4) and Mr. Bayes (codon substitution model) are compared with R package dendextend 1.7.0 [91]. The tangelgram is provided as Additional file 3: Figure S7.

### Gene tree reconciliation

We used Notung [92] for exploring alternate hypotheses about duplication events of the cytokinin signaling genes. Both rooting and a rearrangement tool were used to minimize the overall Duplication/Loss score of a gene tree. A cladogram reflecting land plant evolution was built based on the work of the Angiosperm Phylogeny Group [93] and Zou et al. [94] for relationships between rice species. The most basal eudicot is *Nelumbo nucifera* [95]. *Amborella trichopoda* is sister to all extant angiosperms [96]. Most basal land lineages are represented by the bryophyte *Physcomitrella patens* [97] and *Klebsormidium flaccidium* marking the transition of aquatic to terrestrial life [25]. This cladogram (Fig. 1) was used as a species tree for gene tree reconciliation.

### Comparative genomics (gene collinearity)

We used the PLAZA 3.0 online database [98] to study the genomic organization of the cytokinin signaling genes. In the PLAZA database, collinear and syntenic regions within and between genomes are pre-computed by using i-ADHoRe [99]. An intra-species comparison of the whole genome (WGDotplot) reports all collinear regions, i.e., duplicated blocks, found within a genome. The age of the paralogs is provided based on pairwise synonymous substitution rate (Ks distances) calculated with PAML [100].

## Additional files

Additional file 1: List of analysed species and used data source, and list of the analysed CHK encoding sequences. (XLSX 46 kb)

Additional file 2: Collinear regions, Ks distances. (XLSX 357 kb)

Additional file 3: **Figure S1.** ML Tree CHKs 1, **Figure S2.** Mr. Bayes CHKs, **Figure S3.** Reconciled ML tree CHKs, **Figure S4.** Reconciled ML tree HPTs, **Figure S5.** ML tree RRBs, **Figure S6.** Mr. Bayes tree RRBs, **Figure S7.** Cladogram comparison RRBs. (PDF 2407 kb)

Additional file 4: Supplemental alignment of #CHKs, #HPTs, #RRAs and #RRBs. (FASTA 770 kb)

Additional file 5: RF distances between phylogenetic trees of CHKs, HPTs, RRAs and RRBs. (XLSX 15 kb)

## Abbreviations

CCT domain: Constans/Constans-like/TOC1 domain
CHASE: Cyclases/Histidine kinases Associated Sensing Extracellular
CHK: CHASE domain containing histidine kinase
GDB: Gene dosage balance
GTR: General time reversible (substitution model)
HPT: Histidine phosphotransfer protein
ML: Maximum likelihood
Myb domain: Myeloblastosis related DNA-binding domain
PRR: Pseudo-response regulator
RF: Distance robinson-foulds distance
RRA: Type-A response regulator
RRB: Type-B response regulator
WGD: Whole genome duplication
WGT: Whole genome triplication
