# Supplementary material for: The effects of repeated whole genome duplication events on the evolution of cytokinin signaling pathway
Source: BMC Evol Biol. 2018 May 29;18:76. doi: 10.1186/s12862-018-1153-x (PMC5975490; doi:10.1186/s12862-018-1153-x)
Supplement: Supplementary file 3 — Figure S1. ML Tree CHKs 1, Figure S2. Mr. Bayes CHKs, Figure S3. Reconciled ML tree CHKs, Figure S4. Reconciled ML tree HPTs, Figure S5. ML tree RRBs, Figure S6. Mr. Bayes tree RRBs, Figure S7. Cladogram comparison RRBs. (PDF 2407 kb) [file 12862_2018_1153_MOESM3_ESM.pdf]

Supplemental material

supplemental table 1: list of analyses species and CHK encoding sequences
supplemental table 2: collinear regions, Ks distances
supplemental table 3: RF-distances (tree comparisons)
supplemental fig. 1: ML Tree CHKs 1
supplemental fig. 2: Mr. Bayes CHKs
supplemental fig. 3: reconciled ML tree CHKs
supplemental fig. 4: reconciled ML tree HPTs
supplemental fig. 5: ML tree RRBs
supplemental fig. 6: Mr. Bayes tree RRBs
supplemental fig. 7: Cladogram comparison RRBs

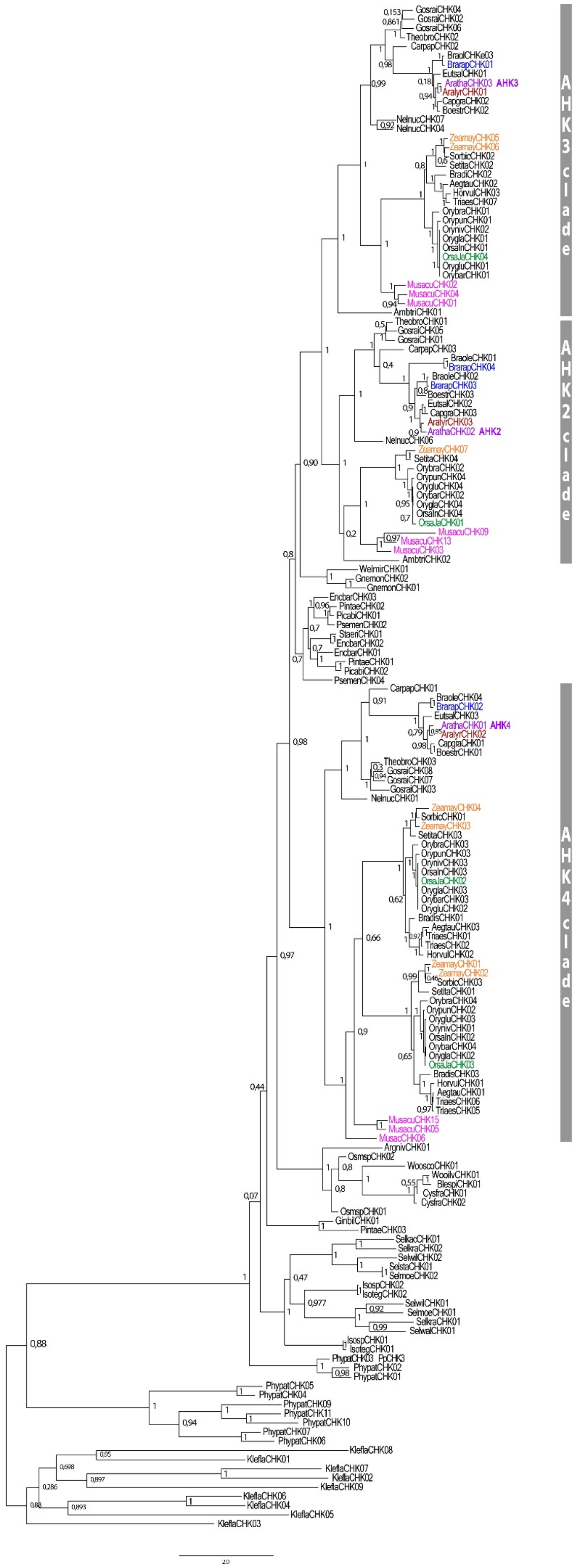

Supplemental fig. 1: **Maximum likelihood tree reconstruction of CHK encoding sequences** (codon substitution model). SH-like branch support is given. Sequences of the core set of investigated plant species are highlighted with the colour code analogous to fig. 2 to fig. 5 in the main manuscript.

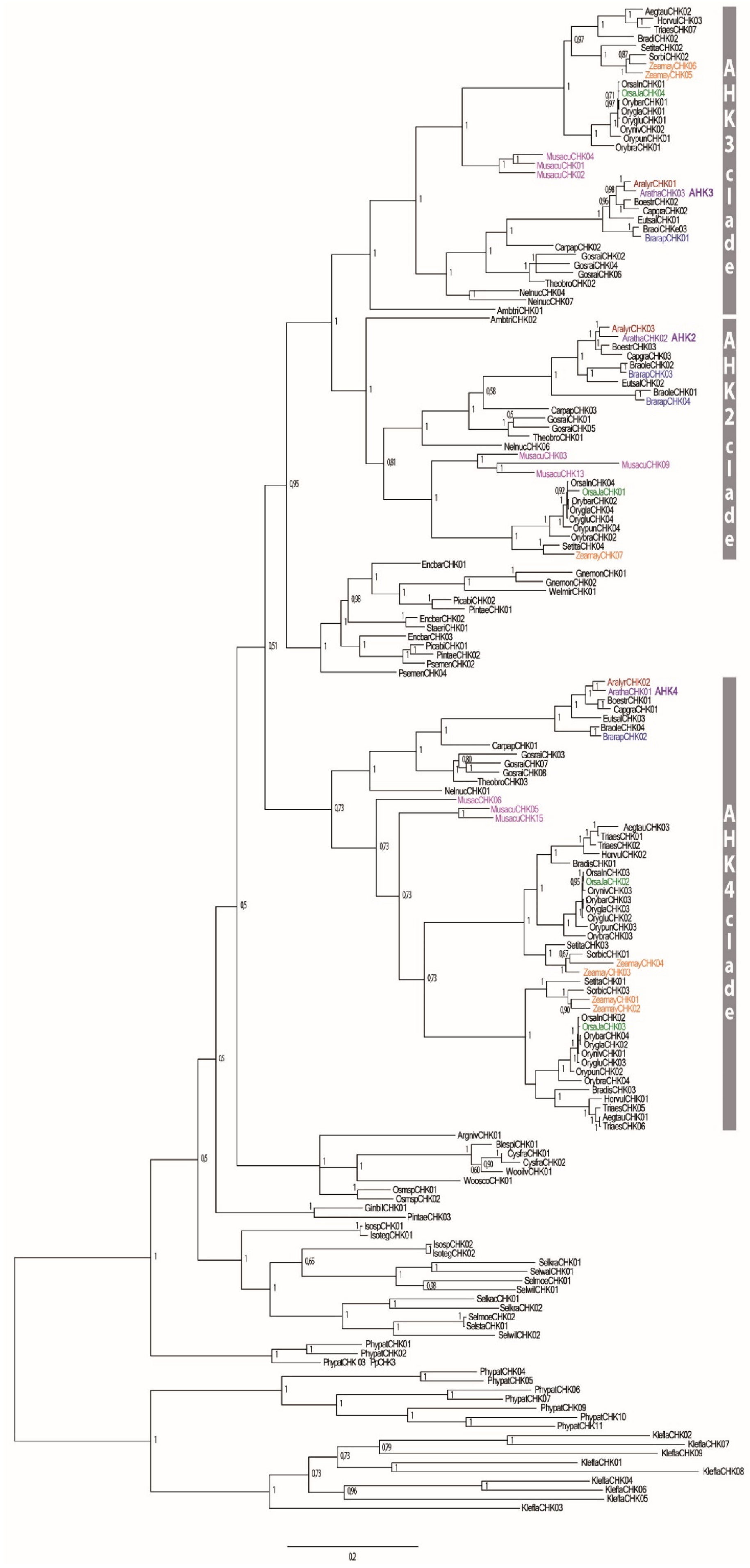

Supplemental fig. 2: **Mr. Bayes tree reconstruction of CHK encoding sequences** (codon substitution model). Posterior probabilities are given. Sequences of the core set of investigated plant species are highlighted with the colour code analogous to fig. 2 to fig. 5 in the main manuscript.

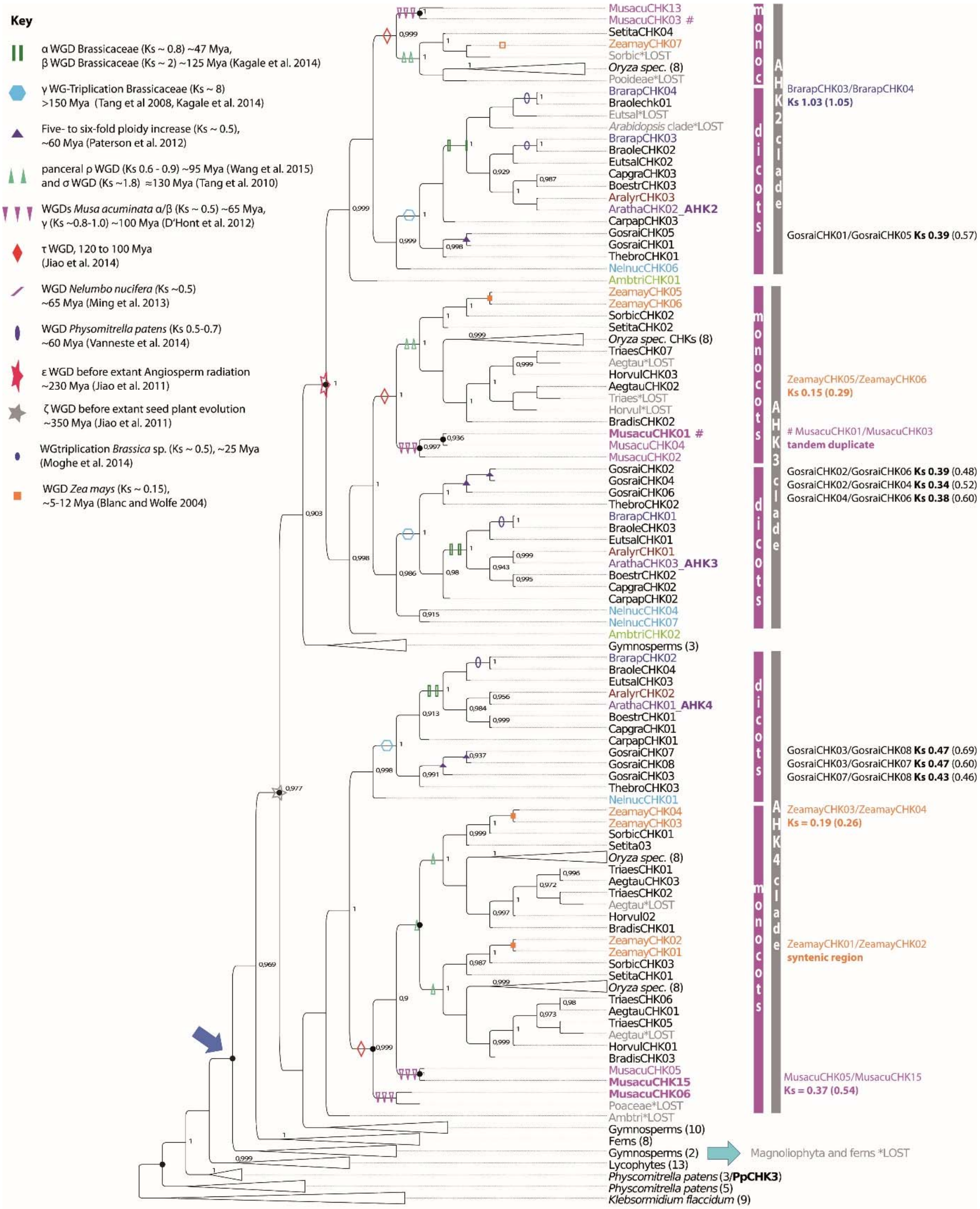

Supplemental fig. 3: Reconciled maximum likelihood tree of CHK encoding sequences (codon substitution model). Gene tree reconciliation included rearrangement of branches with a support less than 0.9 (SH-aLRT branch support). Branch support > 0.9 is given in the tree. Of note, in this reconstructed event history one additional duplication event before the split of gymno- and angiosperms is reconstructed (indicated by a blue arrow) compared to the reconciled Mr. Bayes tree (Fig. 2 main manuscript). Further illustrations are analogous to Fig. 2 in the main manuscript.

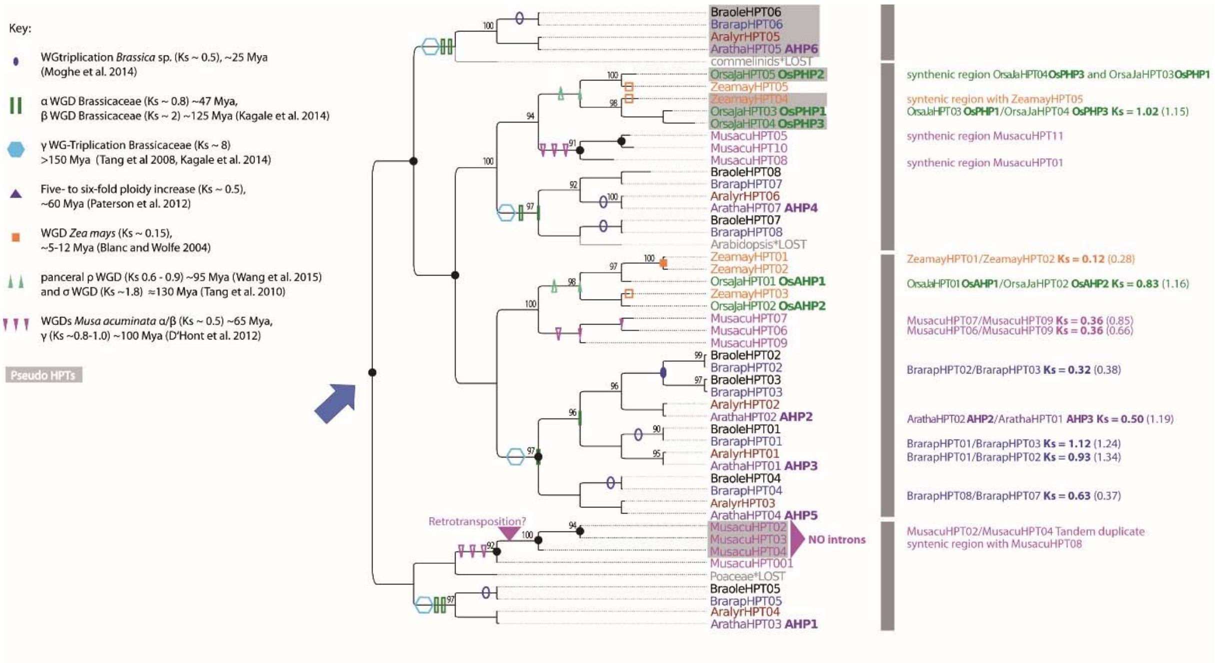

Supplemental fig. 4: Reconciled maximum likelihood tree of HPT encoding sequences (codon substitution model). Gene tree reconciliation included rearrangement of branches with a support less than 0.9 (SH-aLRT branch support). Branch support > 0.9 is given in the tree. Of note, in this reconstructed event history one additional duplication event before the split of mono- and dicots is reconstructed (indicated by a blue arrow) compared to the reconciled Mr. Bayes tree (Fig. 2 main manuscript). Further illustrations are analogous to Fig. 2 in the main manuscript.

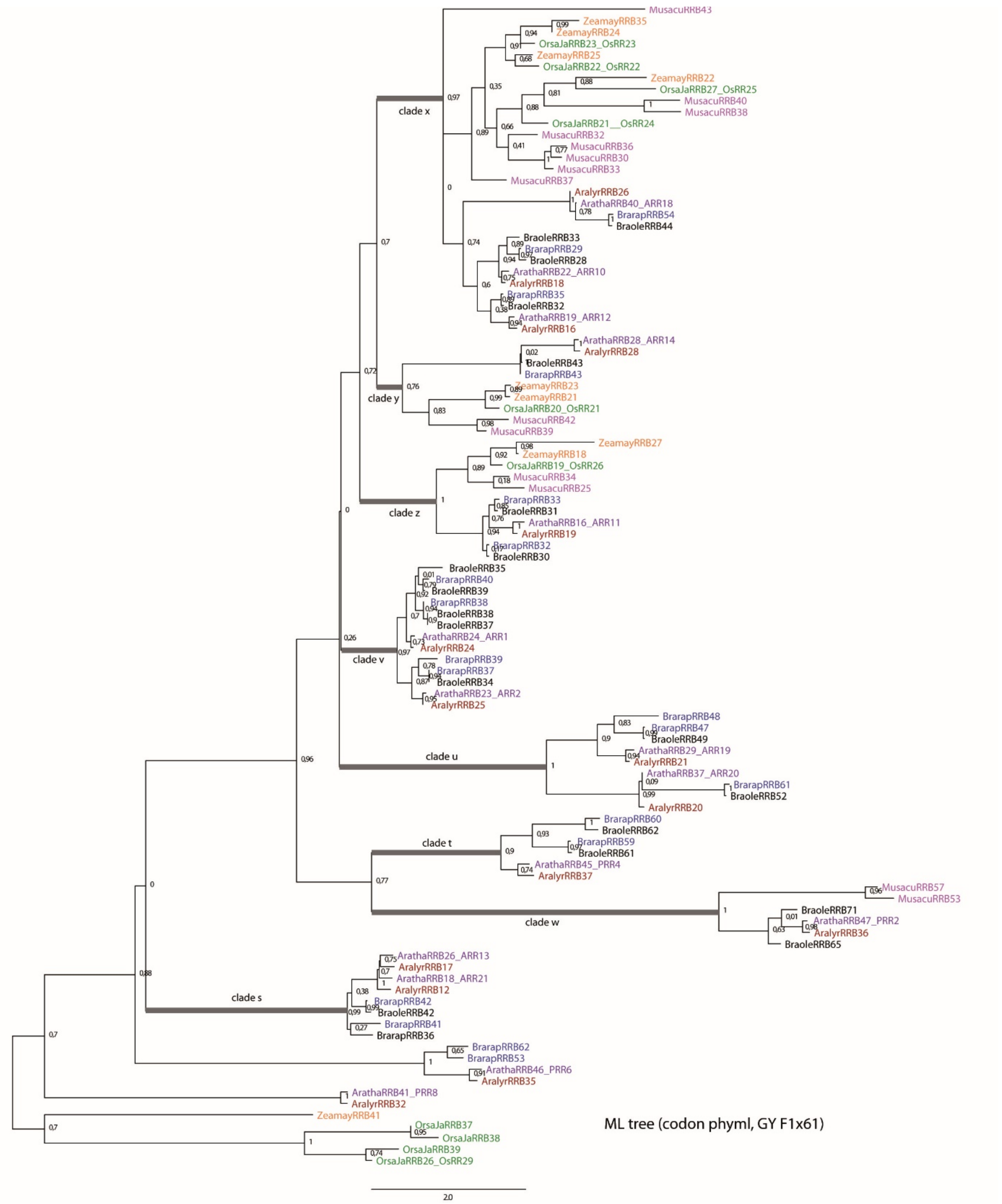

Supplemental fig. 5: **Maximum likelihood tree reconstruction of RRB encoding sequences** (codon substitution model). SH-like branch support is given. Sequences of the core set of investigated plant species are highlighted with the colour code analogous to fig. 2 to fig. 5 in the main manuscript. Branching pattern in Bayesian RRB tree reconstructions differed for basal branches to maximum likelihood tree reconstruction. Groups that are consistently reconstructed in Bayesian and maximum likelihood trees are marked with gray branches.

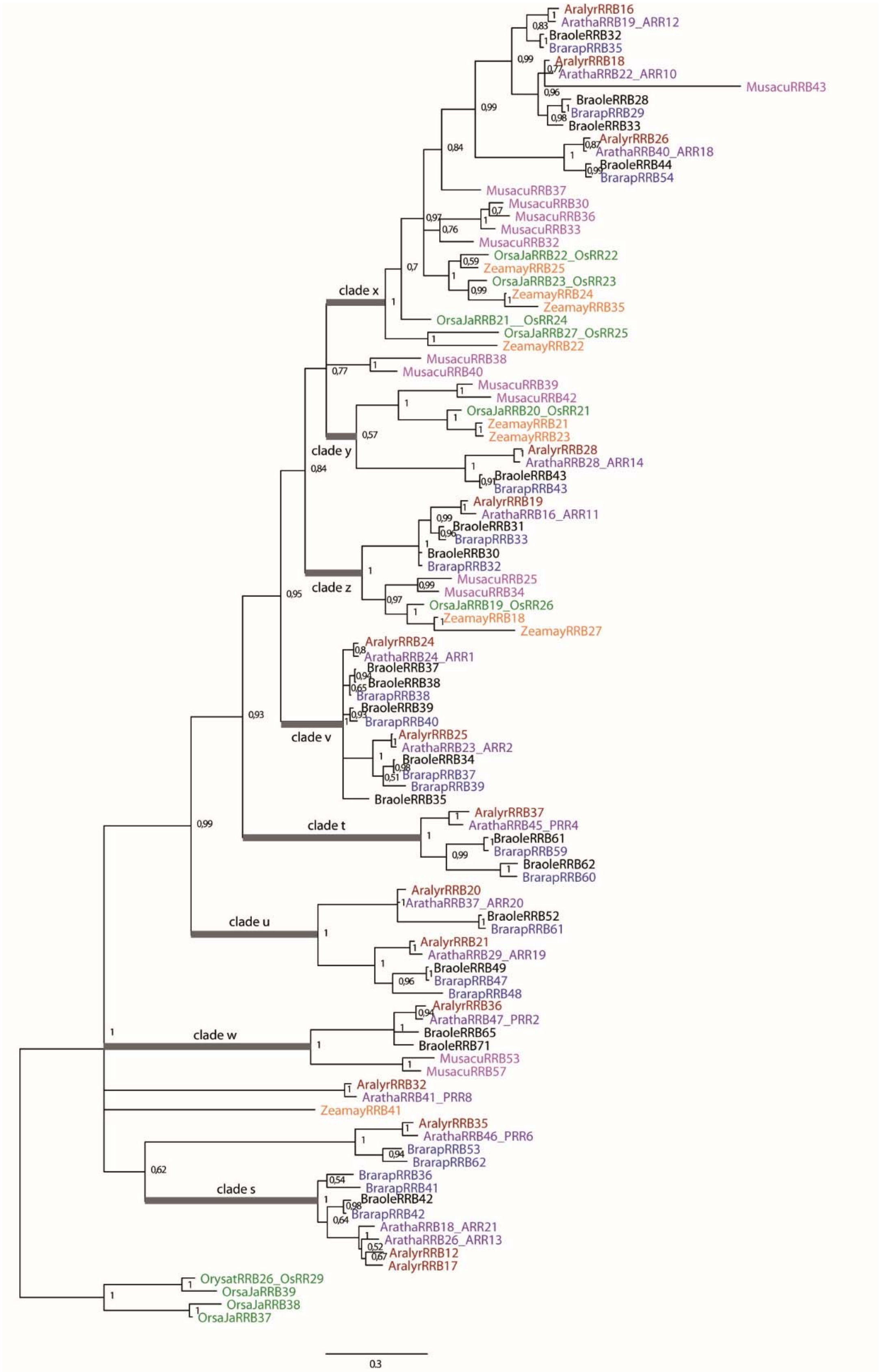

Supplemental fig. 6: **Mr. Bayes tree reconstruction of RRB encoding sequences** (codon substitution model). Posterior probabilities are given. Sequences of the core set of investigated plant species are highlighted with the colour code analogous to fig. 2 to fig. 5 in the main manuscript. Branching pattern in Bayesian RRB tree reconstructions differed for basal branches to maximum likelihood tree reconstruction. Groups that are consistently reconstructed in Bayesian and maximum likelihood trees are marked with gray branches.

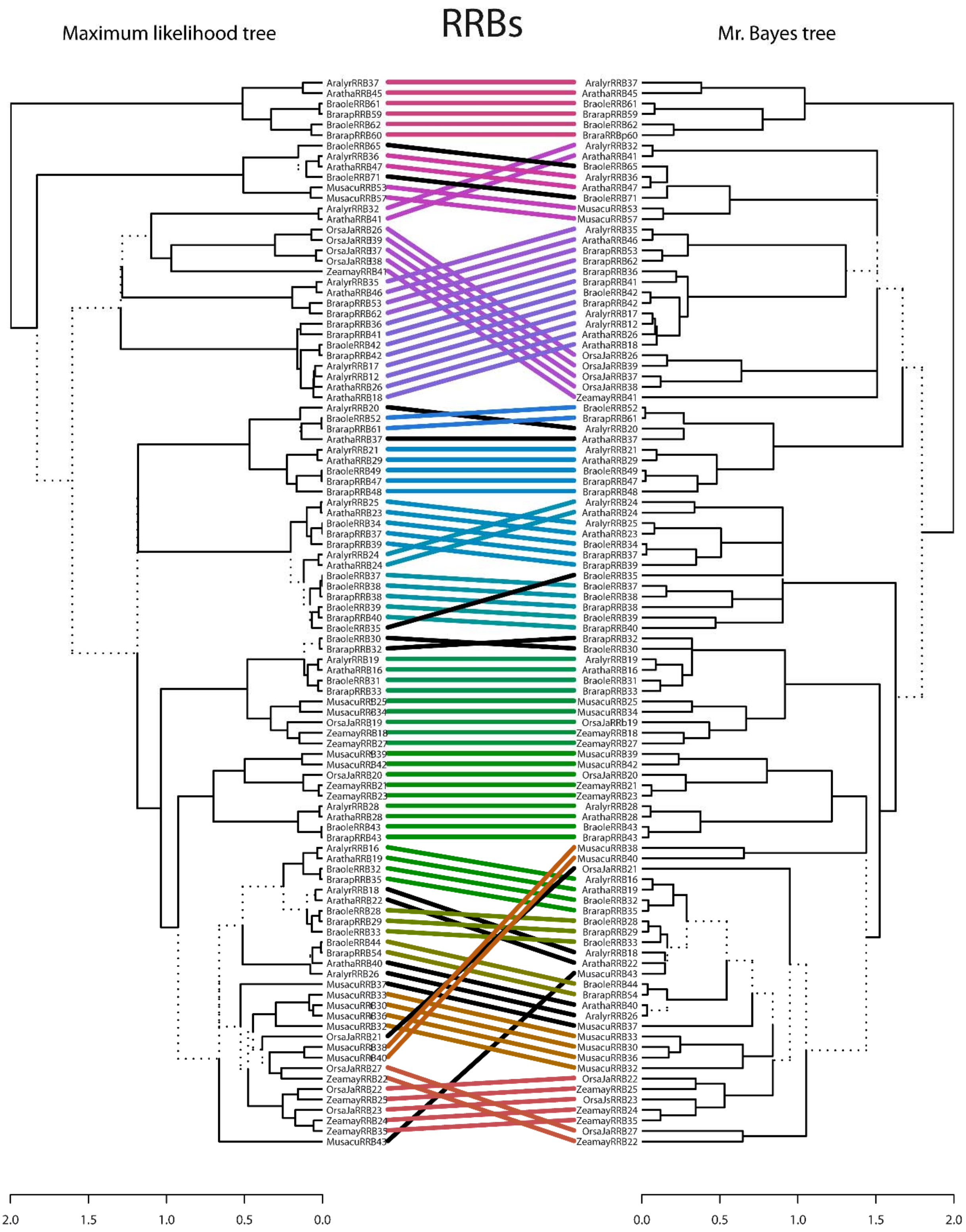

Supplemental fig. 7: **Cladogram comparison RRBs constructed with maximum likelihood** (left, codonphyML, codon substitution model YAP CF3x4) **and Mr. Bayes** (right, codon model). Dotted lines indicate different branches. Colored lines in the middle connect the same species in both trees.
